# Sleep quality, mental health, and cognitive function among older adults in Chinese communities: a cross-sectional study

**DOI:** 10.3389/fpubh.2025.1592886

**Published:** 2025-10-09

**Authors:** Wangsheng Fu, Wei Li

**Affiliations:** ^1^Department of General Psychiatric, The Seventh People’s Hospital of Cixi City, Ningbo, China; ^2^Department of Geriatric Psychiatry, Shanghai Mental Health Center, Shanghai Jiao Tong University School of Medicine, Shanghai, China

**Keywords:** sleep quality, mental health, cognitive performance, community older adults, transverse temporal gyrus thickness

## Abstract

**Background:**

Adequate and good sleep is essential for improving mental health and cognitive function in older adults. However, there is a lack of research on the relationship between sleep and psychiatric symptoms and cognitive function in the Chinese older adult population.

**Methods:**

A total of 621 community older adults aged 60 and older were included in the current study. The Pittsburgh Sleep Quality Index (PSQI), Geriatric Depression Scale (GDS), Self-rating anxiety scale (SAS), and Montreal Cognitive Assessment Scale (MoCA) were administered to all the participants. Based on PSQI, the research subjects were divided into the good sleep quality group and the bad sleep quality group. Moreover, 48 healthy individuals without mild cognitive impairment and dementia also accepted brain MR imaging.

**Results:**

The prevalence of poor sleep was 49.9%, and age, physical exercise, traumatic brain injury as well as family history of sleep disorders were associated with poor sleep (*p* < 0.05). Poor sleepers demonstrated higher prevalence of anxiety and depressive symptoms than good sleeper (*p* < 0.05). In a subgroup analysis of magnetic resonance, individuals with poor sleep quality had higher left and right transverse temporal cortex thickness and higher scores on the GDS and SAS scales. The results of linear regression analysis showed that the total score of SAS was correlated with the thickness of the left transverse temporal gyrus (*T* = 2.115, *p* = 0.042).

**Conclusion:**

About half of the older adults in the community have sleep problems. Poor sleep quality was associated with symptoms of anxiety and depression. Moreover, the cortical thickness of transverse temporal gyrus may be related to anxiety symptoms in older adults with poor sleep quality. This study indicates that a decline in sleep quality may increase the risk of anxiety and depression in patients, and transverse temporal gyrus may play an important regulatory role in the above process.

## 1 Introduction

Sleep-related complaints are so common among older adults that it can be difficult to distinguish whether the complaint is the result of normal aging or a disease process (1). In contrast to newborns, who can sleep 16–20 h a day, many older adults may struggle to get 8 h in one block (2). According to a review article, about 10% of adults suffer from insomnia, another 20% experience insomnia symptoms occasionally, and that figure will rise to 40% for older adults (3). If their sleep is disrupted, they are more likely to suffer from a variety of physical diseases, such as pain, asthma, diabetes, dementia, high blood pressure, immune disorders, physical disability, and gastroesophageal reflux disease (2). In addition, many mental disorders may also be related to sleep disorders. For example, research has found that there is a close correlation between emotional disorders or attention deficit hyperactivity disorder (ADHD) and the type of biological clock (4). A systematic review and meta-analysis indicated that sleep disorders increase the risk of developing dementia (5). Other mental disorders, such as anxiety, depression, and schizophrenia, can also be accompanied by sleep disorders (6–8).

Cognitive function is a high-level neural activity function of the human brain’s functional cortex, which is used to acquire, transform and process external objective information. It mainly covers categories such as memory, calculation, spatial visual ability, and executive ability (9). The main symptoms of cognitive dysfunction include memory, vision impairment, and executive function disorders, while dementia is the ultimate outcome of severe cognitive impairment, which seriously affects the quality of life of patients and increases the burden on society and families (10). Previous studies have shown that sleep disorders can impair the cognitive abilities of the older adult population, which will increase the likelihood of motor and other traffic accidents, industrial accidents, medical errors, and decreased productivity at work (11). What is more serious is that sleep disorders can also increase the risk of developing dementia, for example, in Bloomberg et al. study, they found that suboptimal sleep were independently associated with worse cognitive performance and short sleep was also associated with faster cognitive decline (12).

At present, there are only a few studies on the sleep quality of the older adults community population in China. For example, Liu et al. found that depression moderated the relationship between poor sleep quality and frailty, reducing the relationship by 51.9% (13). In Li et al. study, they found that long sleep duration was significantly associated with poorer mental status and memory scores in Chinese older adults (4). In Li et al. study, they found that suboptimal sleep duration and poor quality were associated with poor cognitive performance in older adults (14). In addition, in Ren et al. study, they also found that sleep duration was associated with all-cause mortality in a J-shaped pattern in the older adult population in China (15). However, almost all the current similar studies were only phenomenological descriptions and seldom involve the exploration of mechanisms, thus greatly reducing the reliability of these studies.

Imaging is one of the most effective tools to study brain structure and function, and is widely used in clinical and scientific research institutions. In Zou et al. study, they found that patients with insomnia disorders showed decreased thalamic connectivity to the left amygdala, parahippocampal gyrus, putamen, pale cortex, and hippocampus during wakefulness and in all three non-REM sleep stages (16). In Bourgouin et al.’s study, they found that idiopathic rapid eye movement sleep behavior disorder (iRBD) patients showed reduced cortical and subcortical gray matter (GM) volume in the caudate nucleus (17). In Ramduny et al. study, they found that inadequate sleep could cause brain ageing, with more atrophy of white and gray matter (18). Moreover, in Zhao et al. (19) and Lee et al.’s (20) study, they also proved that transverse temporal gyrus thickness was associated with different sleep disorders. Thus, hippocampus, amygdala, white matter, gray matter, and transverse temporal gyrus thickness may develop as biomarkers for sleep disorders.

In the current study, we will conduct an investigation and research on the correlation between sleep quality and emotional symptoms among the older adults in Chinese communities. To further explain the related mechanism, we have also included magnetic resonance data. Our hypothesis was that the poorer the sleep quality of the older adults in the community, the more likely they were to have symptoms of anxiety and depression, and had poorer cognitive function. Moreover, it was possible that certain brain structures and cortices, such as the hippocampus, amygdala, white matter, gray matter, and transverse temporal gyrus thickness, played an important role in this process.

## 2 Methods

### 2.1 Participants

The current cohort study was derived from the Brain Health Cohort study in Shanghai,^1^ which has been described in detail in our previous study (21, 22). This cohort included a total of 621 community participants aged 60 years or older. The entry criteria were as follows: (1) aged 60 or more; (2) without severe medical conditions, such as infections, cancer; (3) without serious mental illness, such as severe and depression schizophrenia; (4) be willing to cooperate. Exclusion criteria were as follows: (1) aged below 60; (2) non-permanent residents of Shanghai; (3) impaired vision or hearing and unable to cooperate with the investigation; (4) refusal to cooperate. All eligible participants were required to complete a standard questionnaire, including general information data (e.g., age, education, gender), daily living habits (e.g., smoker, drinker, tea drinker, physical exercise) and disease related information (e.g., hypertension, heart disease, diabetes, hyperlipidemia, surgical history, traumatic brain injury, family history of dementia, and family history of sleep disorders). Moreover, they would also undergo a screening process that included physical and neurological examinations, sleep quality assessment, mental state assessment and cognitive assessment. To explore the mechanism of sleep quality affecting mental state and cognitive function, 48 healthy individuals without mild cognitive impairment and dementia accepted brain MR imaging (poor sleeper *n* = 22; good sleeper *n* = 26).

### 2.2 Sleep quality assessment

In the current study, we mainly used the Pittsburgh Sleep Quality Index (PSQI) to assess subjects’ sleep quality. The Pittsburgh Sleep Quality Index (PSQI) is a tool used to assess two sleep domains: sleep quality and sleep disorders. It has a score range from 0 to 21. A score of 5 or more indicates poor overall sleep quality, while a score of <5 indicates good overall sleep quality (23). A large number of clinical studies have confirmed that PSQI has good sensitivity (89.6%) and specificity (86.5) (kappa = 0.75, *p* < 0.001), which can effectively distinguish between individuals with good sleep quality and those with poor sleep quality (24, 25).

### 2.3 Mental state assessment

Depressive mood and anxiety symptoms were assessed using the geriatric Depression Scale (GDS) and the self-rating Anxiety Scale (SAS). GDS-15 is a useful tool for classifying depression in older adults as mild or severe. It is scored on a scale of 0–15, with a score of 5 or more being considered to have depressive symptoms (26). Similar to GDS, SAS is also a common tool for assessing anxiety symptoms in the older adult population (27). SAS is a norm-referenced scale which enjoys widespread use a screener for anxiety disorders. Its score range is 0–30 points. Generally, a score of 10 or more points is considered to be anxiety symptoms. At present, a number of studies have confirmed that SAS has good sensitivity and specificity, which can be applied in clinical and experimental studies (28).

### 2.4 Cognitive assessment

The Montreal Cognitive Assessment Scale (MoCA) (29) was used to measure the subjects’ overall cognitive function. This screening test consists of 30 items that measure multiple cognitive domains including naming, attention, calculation, abstract, orientation, memory, visual space, as well as language function, and it is one of the most common cognitive assessment tools. Compared with Mini-Mental State Examination (MMSE), MoCA is more sensitive and sensitive to screening for cognitive dysfunction (30).

### 2.5 Magnetic resonance image acquisition and processing

The images were acquired by Siemens Magnetom Verio 3.0 T scanner (Siemens, Munich, Germany), which has been described in detail in our previous article (31, 32). In short, T1-weighted images were obtained from 176 sagittal sections using the three-dimensional magnetization fast gradient echo acquisition sequence. The acquisition parameters were TE = 2.98 ms, TR = 2,300 ms, rotation Angle = 9°, and spatial resolution = 1 × 1 × 1.2 mm^3^. All structural MRI data were processed by using Clinica in FreeSurfer v6.0 (33), in the order of spatial registration, cortical thickness estimation, cortical surface segmentation extraction of subcortical structures, and segmentation into 46 global structures. Then hippocampus, amygdala, white matter, gray matter, and transverse temporal gyrus thickness for each individual were extracted directly using FreeSurfer.

### 2.6 Data analysis

Continuous variables were expressed as mean ± standard deviation(SD), while categorical variables were expressed as frequencies (%). The single-sample Kolmogorov–Smirnov (KS) test was used to test whether these continuous variables conforming to normal distribution. Independent sample t test and non-parametric test were conducted for continuous variables conforming to normal distribution and not conforming to normal distribution, respectively. While Chi-square test (*χ*^2^) was used for categorical variables. Next, a multivariate logistic regression model was used to investigate the association between sleep quality and these possible influencing factors. Then, a general linear model, controlling for age and traumatic brain injury, was used to compare GDS scores, SAS scores, and MoCA scores between the poor and good sleepers. Finally, linear regression analysis was used to investigate the association between mood scores and sleep-related brain regions (the total score of GDS or SAS was used as the dependent variable, and the thickness of transverse temporal gyrus thickness on the left and right were used as the independent variable). Two-tailed tests were used at a significance level of *p* < 0.05 for all the analysis.

## 3 Results

**Figure 1:**
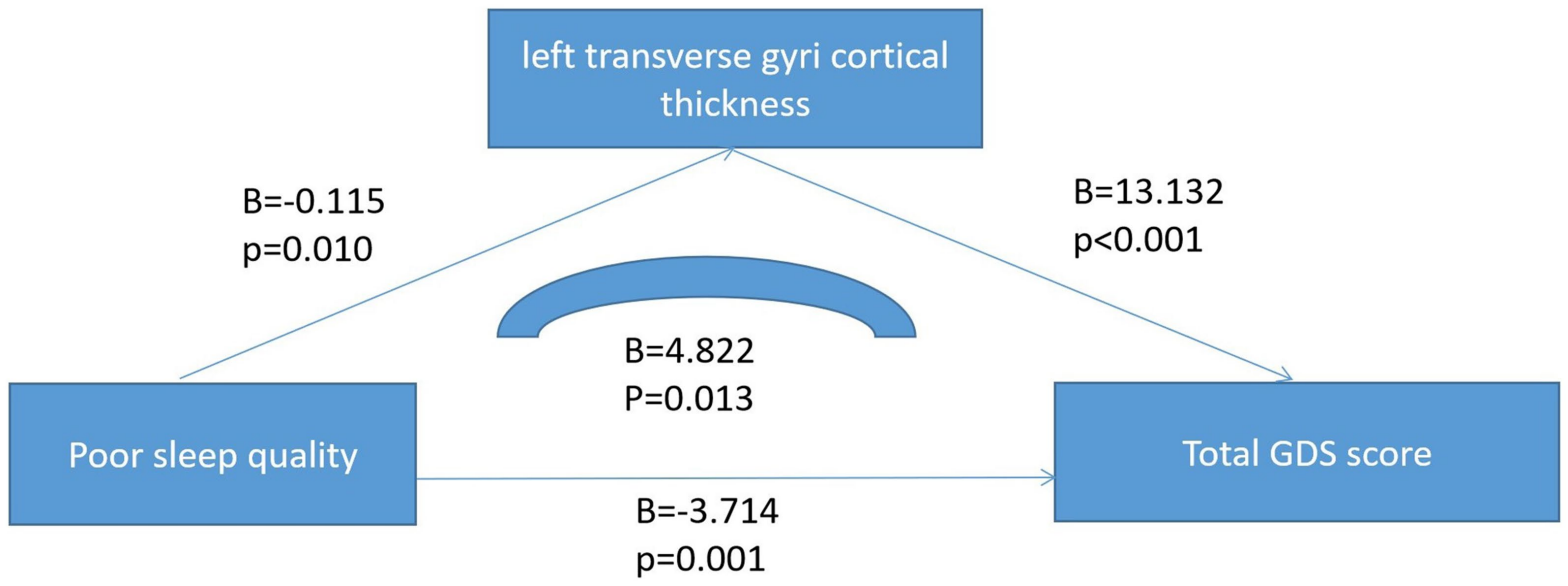
Linear regression analysis of mediation model.

There were statistically significant differences in age and traumatic brain injury (*p* < 0.05) between the poor sleeper group and the good sleeper group (Table 1). Multiple regression analysis showed that age (*B* = 0.025, *p* = 0.024, OR = 1.025, 95% confidence interval: 1.003–1.047) and family history of sleep disorders (*B* = 0.840, *p* = 0.048, OR = 2.317, 95% confidence interval: 1.009–5.322) were associated with poor sleep quality (Table 2). General linear regression analysis showed that poor sleepers had significantly higher GDS and SAS scores than good sleepers (Table 3). Finally, we explored the relationship between emotional scores and sleep-related brain regions, and conducted brain magnetic resonance imaging tests on 48 healthy individuals without mild cognitive impairment and dementia. Based on their sleep quality, we classified them as poor sleepers (*n* = 22) and good sleepers (*n* = 26). Individuals with poor sleep quality had higher left and right transverse temporal gyrus thickness and higher scores on the GDS and SAS scales compared with those with good sleep quality (*p* < 0.05) (Table 4). Linear regression analysis showed that the total score of SAS was positively correlated with the thickness of the left the transverse temporal gyrus (*T* = 2.115, *p* = 0.042). Figure 1 presents the result.

**Table 1:** Comparison of the demographic, lifestyle and cognitive characteristics of good sleepers and poor sleepers among the older adults in the community, based on the Pittsburgh Sleep Quality Index.

Variables	Poor sleeper (*n* = 310)	Good sleeper (*n* = 311)	X^2^ or T	*p*
Age, years	66.69 ± 8.49	65.02 ± 8.51	2.190	0.029*
Education, years	9.65 ± 3.29	10.08 ± 3.29	−1.478	0.140
Male, *n* (%)	112(36.1)	120(38.6)	0.400	0.562
Smoker, *n* (%)	56 (18.1)	69 (22.2)	1.641	0.230
Drinker, *n* (%)	75 (24.2)	66 (21.2)	0.781	0.390
Tea drinker, *n* (%)	131 (42.3)	138 (44.4)	0.283	0.627
Physical exercise, *n* (%)	182 (58.7)	203 (65.3)	2.839	0.099
Hypertension, *n* (%)	135 (43.5)	146 (46.9)	0.723	0.420
Heart disease, *n* (%)	68 (21.9)	56 (18.0)	1.500	0.230
Diabetes, *n* (%)	51 (16.5)	42 (13.5)	1.059	0.314
Hyperlipidemia, *n* (%)	92 (29.7)	76 (24.4)	2.160	0.149
Surgical history, *n* (%)	131 (42.3)	116 (37.3)	1.594	0.219
Traumatic brain injury, *n* (%)	11 (3.5)	3 (1.0)	4.703	0.033*
Family history of dementia, *n* (%)	20 (6.5)	22 (7.1)	0.095	0.873
Family history of sleep disorders, *n* (%)	22 (7.1)	11 (3.5)	3.910	0.051

**Table 2:** The influencing factors of poor sleep quality were investigated by binary logistics regression analysis.

Variables	B	S.E	Wald	df	*p*	OR	95% Confidence interval
Age	0.025	0.011	5.081	1	0.024*	1.025	1.003–1.047
Physical exercise	−0.196	0.189	1.078	1	0.299	0.822	0.568–1.190
Traumatic brain injury	1.301	0.801	2.637	1	0.104	3.673	0.764–17.658
Family history of sleep disorders	0.840	0.424	3.922	1	0.048*	2.317	1.009–5.322

**Table 3:** Comparison of mental health status (anxiety, depression, and cognition) across sleep quality.

Variables	Poor sleeper (*n* = 310)	Good sleeper (*n* = 311)	*F*	*p*
SAS	33.85 ± 7.03	30.32 ± 6.25	7.037	<0.001*
GDS	3.58 ± 2.98	2.22 ± 2.80	6.385	<0.001*
MoCA	22.56 ± 4.71	22.78 ± 4.68	0.547	0.585

**Table 4:** Comparison of brain structure and emotional symptom between poor sleeper and good sleeper.

Variables	Poor sleeper (*n* = 22)	Good sleeper (*n* = 26)	X^2^ or T	*p*
Age, years	71.23 ± 7.58	72.69 ± 4.27	−0.842	0.404
Education, years	8.05 ± 4.59	7.92 ± 3.48	0.107	0.915
Male, *n* (%)	6 (27.3)	9 (34.6)	0.299	0.756
Whole brain volume, cm^3^	1389.12 ± 165.03	1412.92 ± 48.40	−0.702	0.486
Left hippocampus, cm^3^	3.53 ± 0.39	3.51 ± 0.33	0.149	0.882
Right hippocampus, cm^3^	3.76 ± 0.40	3.70 ± 0.46	0.428	0.671
Left amygdala, cm^3^	1.43 ± 0.20	1.45 ± 0.17	−0.421	0.676
Right amygdala, cm^3^	1.64 ± 0.24	1.60 ± 0.17	0.711	0.481
Total gray matter volume, cm^3^	541.28 ± 53.88	542.58 ± 29.60	−0.106	0.916
Total white matter volume, cm^3^	5.93 ± 7.35	3.61 ± 3.60	1.426	0.161
Left transverse temporal gyri thickness, mm^3^	2.29 ± 0.18	2.16 ± 0.19	2.425	0.019*
Right transverse temporal gyri thickness, mm^3^	2.30 ± 0.21	2.16 ± 0.17	2.606	0.012*
SAS	0.34 ± 0.06	0.30 ± 0.06	2.293	0.028*
GDS	5.18 ± 4.42	2.38 ± 3.31	2.505	0.016*
MoCA	23.29 ± 4.28	23.50 ± 4.04	−0.176	0.861

## 4 Discussion

Sleep disorders are a big concept and include insomnia, unconscious sleep, central narcolepsy, hypopnea syndrome, sleep apnea syndrome, central sleep apnea, daytime sleep wake disorder, and restless leg syndrome (34). As one of the most common health problems in today’s society, sleep disorders have a high incidence among the older adults, seriously affecting the physical and mental health and quality of life of the older adults, and even aggravating the condition of the original disease. In the current study, we investigated the relationship between sleep quality, mental state and cognitive function among older adults in Chinese communities, and found that (1) nearly half of the older adults in the community had poor sleep quality; (2) advanced age and a family history of sleep disorders were strongly associated with poor sleep quality; (3) poor sleepers were likely to have more anxiety and depressive symptoms; (4)anxiety symptoms were positively correlated with the left transverse temporal gyrus thickness.

By using the Pittsburgh Sleep Quality Index (PSQI), we found that nearly half of the Chinese older adults in the community had poor sleep quality, and advanced age as well as family history of sleep disorders were major factors in poor sleep quality. Monane et al. (35) estimated that nearly half of people over the age of 65 suffered from insomnia. Moreover, Vitiello et al. also found that older women tend to report sleep disorders more frequently than older men (36), and hormone deficiencies were likely to play an important role in this process (37). So our findings were basically consistent. With the increase of age, the disturbance of sleep rhythm and the reduction of sleep demand often occur, which may lead to the occurrence of sleep disorders. Alternatively, in some patients, insomnia may be caused by an underlying medical condition or a side effect of medication (secondary insomnia) (38). What’s more, genetics may play an important role in the onset and progression of sleep disorders. For example, Lane et al. identified 57 loci for self-reported insomnia symptoms in the UK Biobank and validated their effects in other data (39). Therefore, we need to pay close attention to sleep problems in the older adult population in order to improve their quality of life.

Next, we explored the effects of sleep quality on anxiety symptoms, depressive symptoms, and cognitive function. We found that among older adults in neighborhoods with poor sleep quality, they had higher anxiety scores and depression scores, but there was no difference in overall cognitive function compared to older adults with better sleep quality. In a group of older adults Asian subjects, Yu et al. found that depression and anxiety were associated with some sleep-related problems (40). Among a general population, Matsui et al. found that both poorer sleep quality and shorter sleep duration were associated with poorer mental quality (41). Matsui et al. found that the anxiety symptom clusters were differentially associated with specific sleep-related disorders, highlighting the complex relationship between anxiety and sleep in later life (41). Moreover, McKinnon et al. found that sleep disturbances seemed to predict depressive symptoms (42). So we know with some certainty that sleep problems may be associated with anxiety symptoms and mood symptoms. However, contrary to our expectations, we did not find an association between sleep problems and cognitive function, contrary to the studies by Liao et al. (10) and Guan et al. (43). The reasons may be related to the use of different assessment tools and group differences. Moreover, this might also be related to the fact that we only evaluated the overall cognitive function without specifically evaluating the cognitive function in specific domains. Additionally, when enrolling the above-mentioned population, such as in the study of the second part, we excluded patients with mild cognitive impairment and dementia, and all the subjects enrolled in the study were healthy people. This is also the core reason for the lack of cognitive differences.

Finally, we explored the links between sleep quality, mood symptoms, and brain structure. Through linear regression analysis, we found there was a positive correlation between the total score of SAS and the cortical thickness of the left transverse temporal gyrus. The transverse temporal gyrus, also known as the Heschl gyrus or Heschl cyclotron, is part of the larger region of the superior temporal gyrus. It includes the lower two-thirds of the primary auditory cortex, and the anterolateral subregion, which is important for pitch processing (44). In Jin et al.’s study, they found that the transverse temporal gyrus thickness was associated with posttraumatic stress disorder (PTSD) in patients with childhood trauma (45). In Qi et al. study, they found that the transverse temporal gyrus thickness was associated with PTSD symptom severity in Han Chinese adults who lost their only child (44). Moreover, in Cruz-Gomez et al. study, they also found that the right superior and transverse temporal gyri was associated with anxiety symptoms in patients with multiple sclerosis (MS) (46). Based on our and others’ research conclusions, we speculate that the cortical thickness of transverse temporal gyrus may be related to anxiety symptoms and may be a new therapeutic target for anxiety symptoms, but the above conclusions need to be further verified.

We must admit that our study has several limitations. Firstly, this was only a cross-sectional study, so it could not establish a causal link between sleep quality and mood symptoms; Secondly, the magnetic resonance data was relatively small, which might cause some deviation to the research results. Finally, the lack of assessments of cognitive function in specific areas may have influenced the results.

## 5 Ethics and data approval

This study was approved by the Ethics Committee of Shanghai Mental Health Center, and all subjects signed informed consent before the study began. The whole study was carried out in accordance with the principles of the Declaration of Helsinki.

## 6 Conclusion

Nearly half (49.9%) of the older adults in Chinese communities suffer from poor sleep quality, which can lead to symptoms of anxiety and depression. In addition, the cortical thickness of transverse temporal gyrus (*T* = 2.115, *p* = 0.042) may be related to anxiety symptoms in older adults with poor sleep quality.

## Data Availability

The original contributions presented in the study are included in the article/supplementary material, further inquiries can be directed to the corresponding author.

## Footnotes

^1^ http://www.shanghaibrainagingstudy.org/
